# Dysregulation of *miR-122*, *miR-574* and *miR-375* in Egyptian patients with breast cancer

**DOI:** 10.1371/journal.pone.0298536

**Published:** 2024-05-31

**Authors:** Eman A. Elghoroury, Esmat E. Abdelghafar, Solaf Kamel, Eman Awadallah, Aliaa Shalaby, Gamila S. M. EL-Saeed, Eman Mahmoud, Mahmoud M. Kamel, Asmaa Abobakr, Rasha Nazih Yousef

**Affiliations:** 1 Department of Clinical & Chemical Pathology, Medical Research and Clinical Studies Institute, National Research Center, Giza, Egypt; 2 Medical Biochemistry, Medical Research and Clinical Studies Institute, National Research Center, Giza, Egypt; 3 Clinical Pathology Department, National Cancer Institute, Cairo University, Cairo, Egypt; 4 Baheya Centre for Early Detection and Treatment of Breast Cancer, Giza, Egypt; Tanta University Faculty of Medicine, EGYPT

## Abstract

**Background:**

The early detection of breast cancer (BC) is receiving global attention, creating an urgent need for more sensitive and comprehensive strategies for preventive intervention, therapy assessment, and prognosis prediction. Aberrant expression of miRNAs has been observed in various malignancies and may be potential targets for therapy. Our study aims to examine the expression profiles of *miR-375*, *miR-574-3p*, and *miR-122* in the sera of Egyptian women with BC, benign breast lesions, and a control group. We hope to determine if these miRNAs can serve as minimally invasive biomarkers for BC.

**Methods:**

This is a case-control study in which 77 patients with newly diagnosed BC, 20 patients with benign breast tumors, and 30 normal healthy subjects as controls were recruited from the outpatient clinic of the National Cancer Institute. The assessment of miRNAs was conducted using RT-PCR (Applied Biosystems).

**Results:**

The expression level of *miRNA-122* was significantly upregulated in the BC group, while the expression levels of *miRNA-574* and *miRNA-375* showed significant downregulation in BC patients. Serum *miR-122* and *miRNA-375* were able to distinguish breast cancer from the benign and control groups in ROC curve analysis, with AUCs of 0.786 and 0.796, respectively. Our results also showed that serum *miR-122* and *miR-574* are significant predictor variables in the multivariate analysis, after adjusting for age.

**Conclusions:**

Our findings suggest that *miR-122* may act as an onco-microRNA, while *miR-574* and *miR-375* may have a main tumour suppressor role. The studied miRNAs may serve as minimally invasive biomarkers for cases of breast cancer and as promising potential therapeutic targets for breast cancer.

## Background

In Egypt, breast cancer (BC) is the most prevalent form of cancer among women, making up 38.8% of all cases. It is estimated that there were about 22,700 cases of BC in 2020, and this number is expected to rise to about 46,000 by 2050. BC is the second leading cause of cancer-related deaths among women globally [1]. In Egypt, the average age for BC diagnosis is 50.4 years, with 57% of cases occurring in premenopausal or perimenopausal women. In comparison, the median age at diagnosis in Japan is 59.7 years and, in the US, it is 63 years, according to the Japanese Breast Cancer Society registry and US-based SEER data [2].

The age distribution in Egypt may be explained by the population pyramid, with only 8–9% of females over 60 years old. Additionally, 20% of BC patients in Egypt were under 40 years old, compared to 5% in SEER. In developed countries like Korea, the population of premenopausal females has decreased over time, from 59.4% in 2002 to 46.5% in 2015 [2]. Early detection of BC is gaining worldwide attention, so there is an urgent need to find more sensitive and comprehensive strategies for preventive intervention, assessment of therapy, and prediction of prognosis [3, 4].

MicroRNAs (miRNAs) are small non-coding RNAs that regulate gene expression. They can be secreted into extracellular fluids and transported to target cells via vesicles like exosomes or by protein binding. Extracellular miRNAs act as chemical messengers, mediating cell to cell communication [5]. They base pair with 3´untranslated region of the messenger RNA to be suppressed, causing halting, or slowing of the translation process. This step is essential for the targeted proteins production regulation [6]. Due to their multi-level regulatory action, miRNAs have attracted significant interest in the field of cancer research. Depending on the target gene that they regulate, miRNAs can act as ’ tumour suppressor miR ’ by suppressing oncogenes or ’onco-miR’ by targeting tumor suppressor genes [7].

Abnormal miRNA expression has been found in different types of cancer, suggesting that they may be important in the development and progression of tumour. Additionally, miRNAs are easy to isolate, structurally stable, and not affected by sample isolation and processing, making them potential diagnostic and prognostic markers, as well as targets for therapy [8]. miRNAs expression levels are altered in BC due to several mechanisms including transcription factors, epigenetic factors, and protein mutations [9].

*MiR-375* is beta cell-specific miRNA located on *Chr2* (*219*,*001*,*648–219*,*001*,*669*), it serves as a multifunctional regulator in various cellular pathways. Aberrant expressions of *miR-375* are commonly associated with pathological changes [10]. It has been suggested that it may act as a tumour suppressor or oncogene in different types of tumours, but its expression and regulatory role in breast cancer (BC) are still not fully understood [11]. *MiR-574-5p* play an important role in regulating the migration of tumour cells, including those in BC, thyroid cancer, non-small cell cancer, and colorectal cancer [12]. *miR-574-3p* was previously identified as a potential prognostic marker for BC [13]. It has been found to suppress proliferation, migration, and epithelial mesenchymal transition in triple negative BC cells. Additional research has confirmed that *MiR-574-5p* reduces both tumour size and metastasis in vivo [13].

*MiR-122* was initially identified as a tumour suppressor gene in hepatocellular carcinoma. It can suppress the expression of *SRF* and *ADAM10*, which play important roles in the development of various cancers. Over-expression of *miR-122* may promote cell death and halt the cell cycle in cancer cells by reducing the expression of *Bcl-W* and/or *CCNG1*. These findings indicate that *miR-122* may act as a tumour suppressor in cancer [14].

In BC, *miR-122* has been found to act as both a tumour suppressor, targeting the insulin-like growth factor 1 receptor, and as an oncogene, reprogramming glucose metabolism in the tumour microenvironment via exosome [15]. The role of *miR-122* has been considered both as tumor suppressor miRNA and onco miR in breast tumor phenotypes and *miR-122* may also increase the sensitivity of tumor cells to chemotherapy agents [15].

However, there is a lack of sufficient data on the expression of miRNAs and their role in Egyptian women with BC. Therefore, the objective of the present study was to examine the expression profiles of *miR-375*, *miR-574-3p*, and *miR-122* in the sera of Egyptian women with BC, benign breast lesions, and a control group. The aim is to determine if these miRNAs could potentially serve as minimally invasive biomarkers for BC.

## Methods

### Characteristics of patients

This case control study included 77 consecutive patients with newly diagnosed BC who were recruited from outpatient clinic of National Cancer Institute, Cairo University and Baheya Centre of Early Detection and Treatment of Breast Cancer, Giza, Egypt between February 2020 and March 2021, 20 patients with breast benign breast lesions and 30 normal healthy subjects as controls (age and sex matching). None of the healthy controls had been previously diagnosed with any malignancy. All the recruited BC patients have been pathologically diagnosed as BC.

#### General Inclusion criteria

Primary single malignancy and Subject age range 21–65

#### Exclusion criteria

Age <21 or >65, patients received chemotherapy.

This study was approved by the National Research Centre Ethics Committee on 16-9-2019 (Approval No. 19–206) and is in accordance with the ethical standards of the Declaration of Helsinki and written informed consent was obtained from all participants.

Extraction of serum miRNAs was done using miRNA assay Serum/Plasma Kit (Qiagen, Hilden, Germany). All serum RNA preparations were quantified by NanoDrop 1000 (Nanodrop, Wilmingtion, Delaware, USA). cDNA was done using TaqMan microRNA Reverse Transcription Kit (Applied Biosystems, Carlsbad, CA, USA) and microRNA-specific stem-loop primers (part of the TaqMan microRNA Assay Kit; Applied Biosystems). Real-time Quantitative PCR was performed using the Rotor gene Q Real Time PCR System (Qiagen, Valencia, CA, USA).

We selected miRNAs that had been shown to be dysregulated in BC. Researchers have found significant up regulation of *miR-574-3p* acting as a tumour promoter in breast cancer, osteosarcoma, and gastric cancer [16]. Cardinali B. and colleagues revealed the diagnostic potential of the seven miRNAs (*miR-375*, *miR-21-5p*, *miR-205-5p*, and *miR-194-5p*, upregulated, and *miR-376c-3p*, *miR-382-5p*, and *miR-411-5p*, downregulated), as well as (HR+) BC patients, suggested recurrence in TNBC [17]. *MiR-122a*, *miR- 191*, *miR-382-5p*, and *miR-213* had increased expression in a study on the aberrant expression of miRNAs in ER-positive breast cancer, while *miR-145*, *miR-125b-1*, and *miR-125b-2* had decreased expression levels [18].

The relative expression levels of *miR-375*, *miR-574-5p*, and *miR-122* were calculated and normalized to miR-16 (Applied Biosystems, Foster City, CA) using 2^-ΔΔct^ method [19]. Normalization is a key process in quantitative analysis of miRNA levels by qPCR. MiR-16 showed little overall and between-group variability and seemed to be dysregulated in myelodysplastic syndrome and many cancer types [20], so we used *MiR-16* as a standard miRNA to compare expression levels of other miRNAs in plasma of studied groups.

### Statistical analysis

Data were analysed using SPSS win statistical package version 24. Numerical data will be expressed as mean and standard deviation (SD), median and range as appropriate. Qualitative data will be expressed as frequency and percentage. Chi-square (Fisher’s exact) test will be used to examine the relation between qualitative variables as appropriate.

Multivariate analysis was done for variables statistically significant on univariate level to indicate independent predictive factors using logistic regression model. Calculation of sensitivity, specificity, positive predictive value, negative predictive value and total accuracy with their 95% confidence interval will be done. Correlation analysis was done by using Pearson correlation. P-value ≤0.05 will be considered statistically significant and all tests will be 2 tailed.

## Results

Our study included 77 patients with primary BC, 20 patients with benign breast lesions, and 30 controls. Descriptive data for the BC group are presented in Table 1. We found no significant differences between the groups studied in terms of age or hormone receptor status, including *ERs*, *PRs*, *and HER2*.

**Table 1:** Baseline characteristics of BC group (n = 77).

	N	%
Age		
Mean ± SD	52.92 ± 10.953	
Median (IQR)	53.00 (29.0–75.0)	
< = median value	39	50.6
>median value	38	49.4
Histopathology pattern		
ductal carcinoma insitu	3	3.9
Invasive duct carcinoma	62	80.5
Invasive lobular carcinoma,	6	7.8
Invasive micro papillary carcinoma	1	1.3
Invasive tubular/cribriform carcinoma	1	1.3
mixed invasive duct and invasive lobular carcinoma	1	1.3
Mucinous carcinoma	3	3.9
Grades		
grade I	5	6.5
grade II	52	67.5
grade III	17	22.1
Grade 0	3	3.9
**ER status**		
negative	2	2.6
positive	75	97.4
**PR status**		
negative	2	2.6
positive	75	97.4
**HER2 status**		
negative	65	84.4
positive	12	15.6

The expression level of *miRNA- 122* was significantly up regulated in BC group than in the benign breast lesions and control groups: those of patients with benign breast lesions and controls however, on analysing the expression levels of *miRNA 574* and *miRNA 375*, patients with BC showed significant down regulation in comparison to the benign breast lesions and control groups as shown in Table 2. Relation Between miRNA 122,574, and 375 and Clinicopathologic Features of BC Patients were presented in S1 Table.

**Table 2:** Expression of miRNAs among studied groups.

Group		*mir122*	*mir574*	*mir375*
BC	Median (IQR) (Min -Max)	8.0 (1.0–64.0)	0.006 (0.0003–0.45)	0.1250 (0.0010–1.0)
Other groups	Median (IQR) (Min -Max)	2.0 (0.0078–32.0)	2.0 (0.0006–64.0)	4.0 (0.0020–128.0)
P value		**0.002**	**<0.001**	**<0.001**

With an AUC of 0.786, serum *miR-122* was able to distinguish the benign breast lesions and control groups from BC in ROC curve analysis, with CI = 0.699 to0.873, p <0.001, with sensitivity = 76.6%, specificity = 70%, at a cut-off 2.5 The analysis of ROC curve also revealed that serum *miR-574* discriminated the benign breast lesions and control groups from BC with AUC = 0.800, 95% CI = 0.708 to 0.893, p < 0.0001, with sensitivity = 74%, specificity = 75.3%, at a cut-off 0.0139. Serum *miRNA-375* also distinguished BC from benign breast lesions and control groups with AUC = 0.796, 95% CI = 0.711to0.882, p < 0.001, with sensitivity = 70%, specificity = 68.8%, at a cut-off 0.1875 Fig 1. In addition to ROC curve analysis, multivariate logistic regression was done to select the independent predictor factors associated with BC risk among non-malignant groups. Our results showed that serum *miR-122* and *miR-574* are considered as significant predictor variables in the multivariate analysis, with adjustment for age as shown in Table 3.

**Fig 1:**
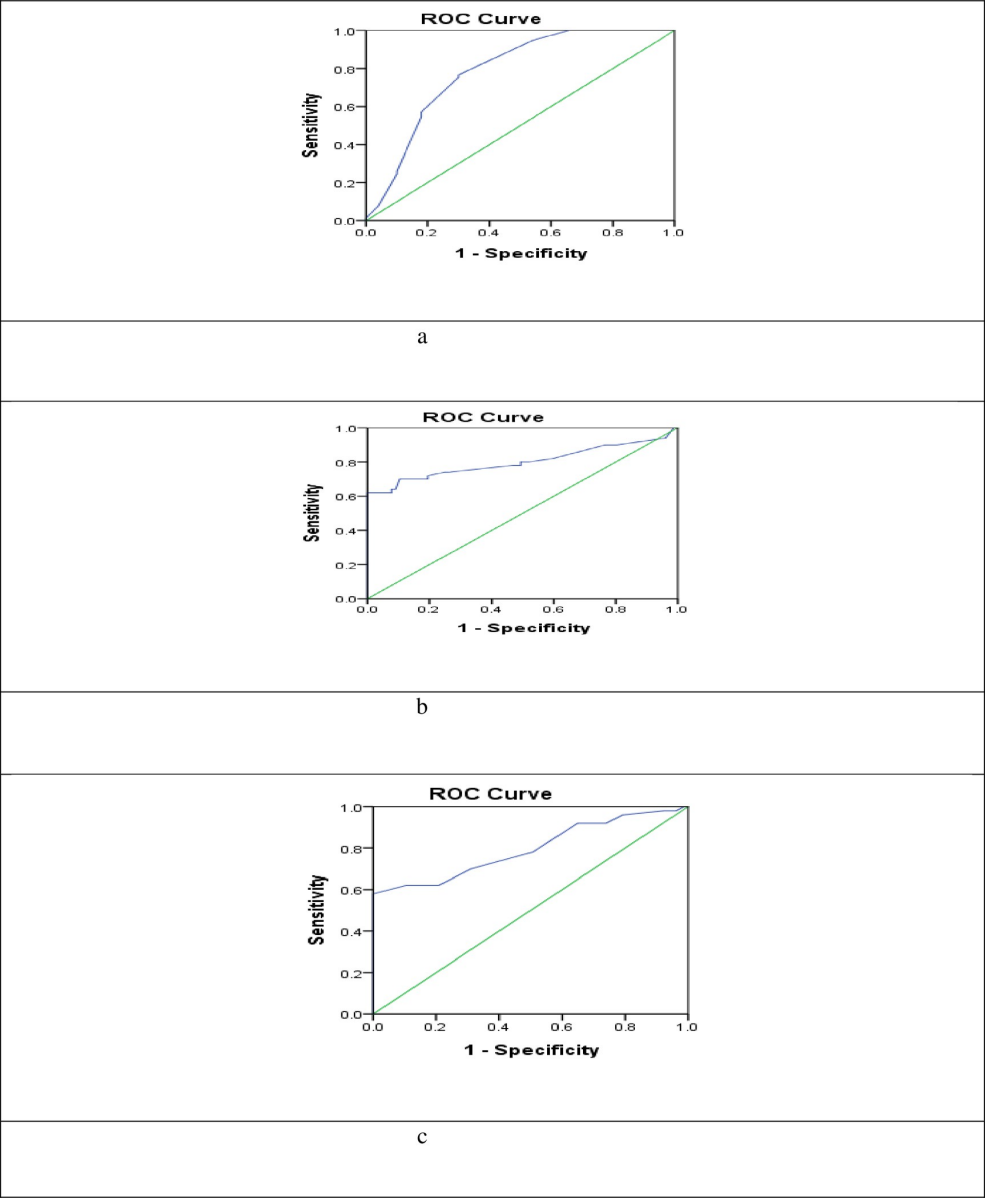
Diagnostic accuracy of serum mir122, mir574 and mir375. A: ROC curve of mir122, B: ROC curve of mir574, C: ROC curve of mir575.

**Table 3:** Multivariate analysis (logistic regression) model.

Independent predictive factor	Beta coefficient	Standard error	p value	odds ratio	95% C.I. for odds ratio	
					Lower	Upper
*mir122* (>2.5 v. < = 2.5)	1.432	0.463	0.002	4.185	1.689	10.371
*mir574* (< = 0.0139 v.>0.0139)	1.560	0.474	0.001	4.758	1.880	12.041
*mir375* (< = 0.1875 v >0.1875)	0.552	0.489	0.258	1.737	0.667	4.527

In BC, we found no significant association between *miRNAs 122*,*574* and *375* and age subgroups (P = .236, .427, and .092 respectively). No significant difference was found in our study between hormone receptor status and studied miRNAs (P = .597, 334 and .497) for estragon receptors, (P = .586, .482, and.831) for progesterone receptors, and (P = .482,.694, and.301) for human epidermal growth factor receptor 2.

**Fig 2:**
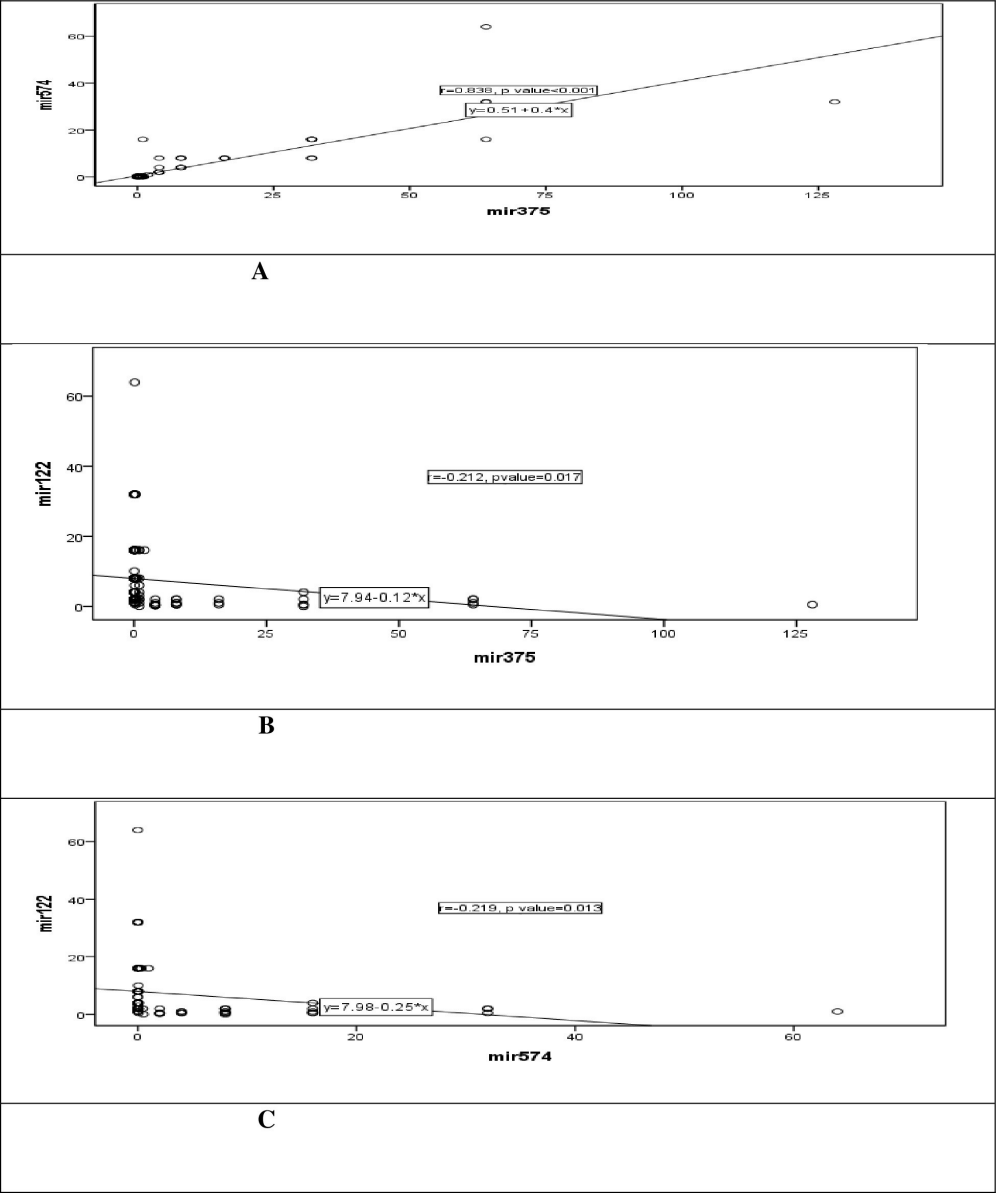
Scatter diagram showing linear relation between mir122, mir574 and mir375. A: linear relation between mir375 & mir3574, B: linear relation between mir122 & mir375, C: linear relation between mir122 & mir574.

Spearman correlation analysis showed a positive significant correlation between *mir574* and *mir375* (p<0.001) and weak negative significant correlation between *mir574* and *mir122* (p = 0.013) and *mir375* and *mir122* (p = 0.017) as shown in Table 4, Fig 2.

**Table 4:** Correlation analysis between studied miRNAs.

Pearson correlation		*mir574*	*mir375*
***mir375***	Pearson Correlation coefficient	0.838	
	p value	**<0.001**	
***mir122***	Pearson Correlation coefficient	-0.219	-0.212
	p value	**.013**	**.017**

## Discussion

The incidence of BC is rapidly increasing in developed countries. Early detection not only improves treatment outcomes, but also has a positive impact on the psychological, economic, and social complications of this disease [21]. Therefore, there is a significant focus on finding minimally invasive, cost-effective diagnostic biomarkers for BC. This research aims to identify and investigate their utility for diagnosing and prognosing different cancers, including BC [22]. Several miRNAs have appeared in the plasma or serum of patients with BC. MiRNAs profiling studies can categorize dysregulated miRNAs and group BC patients for treatments, this may demonstrate the potential of miRNA as a prognostic and therapeutic biomarker [23].

In BC patients, *miR-122* has been identified as a tumour suppressor that targets the insulin-like growth factor-1 receptor [24]. A previous study showed that *miR-122* acts as a tumour suppressor by regulating oncogenes such as *cyclin G1*, *AKT3*, *and CDK4* in hepatocellular carcinoma [25]. Downregulation of *miR-122* has been observed in hepatocellular carcinoma [26].

On the other hand, higher levels of circulatory *miR-122* correlate with hepatocellular carcinoma [27] and liver injury [28]. Previous study reported that *miR-122* Expression has been detected in primary fibroblasts, where it is involved in *p53mRNA* polyadenylation/translation by targeting cytoplasmic polyadenylation element binding protein [29].

A study by Fong et al. found that breast cancer (BC) secretes an excess amount of *miR-122*, suggesting that it may act as an onco-microRNA [30]. The physiological role of *miR-122* differs according to the type of cancer [19]. Our study demonstrated a significantly higher expression of serum *miRNA122* in BC patients compared to age-matched patients with benign breast lesions and healthy controls. To find a possible relationship between *miRNA 122* and the clinicopathological features of BC patients, we did not find any association between *miR-122* and any of these features. However, a study by Wu et al. found that higher levels of *miR-122* were associated with *HER2-*negative and non-inflammatory tumours [31]. This difference could be explained as most of our patients were *HER2-*negative.

A study by Zhang et al. found that overexpressing *miR-574-5p* significantly reduced tumor weight and size in vivo. Additionally, *miR-574-5p* overexpression inhibited pulmonary migration and significantly decreased the number of metastatic nodules. This suggests that *miR-574-5p* may prevent tumorigenesis and lung metastasis in vivo [13].

Our BC patients exhibited downregulation of *miR-574*, indicating its role as a tumour suppressor miRNA. *MiR-574* inhibits *SKIL* expression by targeting *SOX2 and BCL11A*, thereby inhibiting proliferation, migration, and epithelial-mesenchymal transition (EMT) in triple negative BC [32]. Similarly, *miR-375*, which acts as a tumour suppressor miRNA, is dysregulated in numerous cancers [33].

The results of this study revealed a downregulation of *miR-375* in the BC group, which is consistent with previous research that also reported a downregulation of *miR-375* in BC [34, 35]. However, some studies have indicated that *miR-375* is significantly upregulated in BC [36, 37]. Therefore, the expression level of miR-375 in BC tissues remains controversial, and further studies and research are needed to clarify its role in BC. Additionally, the clinical significance and potential targets of *miR-375* in BC have not been comprehensively investigated thus far. The discrepancy between results may be attributed to differences in sample sizes and types.

Study by Li,L et al. revealed that *miR-375* was significantly downregulated in HCC cell lines and tissues compared with normal hepatic cells and tissues. Further investigation by Li,L et al showed that the stimulation of *miR-375* can supress cell proliferation of HCC and stimulate apoptosis. Thus, *miR-375* regulation has key role in tumorigenesis of HCC *in vitro* [38]. Previous research have shown that upregulation of *ErbB2* gene contributes significantly to hepatocellular growth, and the upregulation of *ErbB2* was associated with the regulation of *miR-375* [39].

In our ROC analysis, serum *miR-122* had an AUC of 0.786 and was able to distinguish between benign breast lesions and control groups from BC with a sensitivity of 76.6% and specificity of 70% at a cut-off of 2.5. The analysis of the ROC curve also revealed that serum *miR-574* discriminated between benign breast lesions and control groups from BC with an AUC of 0.800 at a cut-off of 0.0139, with a sensitivity of 74% and specificity of 75.3%. Serum *miRNA-375* also distinguished BC from benign breast lesions and control groups with an AUC of 0.796, a sensitivity of 70%, and a specificity of 68.8% at a cut-off of 0.1875.

### Limitations of the study

The main limitation of this study was the lack of a power analysis to calculate the sample size. Additionally, due to budget constraints, we were unable to have an equal number of participants in all groups.

## Conclusion

In conclusion, we found that serum *miRNA-122* is upregulated in patients with BC, while *mir-574* and *miR-375* expression are downregulated. Our findings suggest that *miR-122* may act as an onco-microRNA, while *mir-574* and *miR-375* have a tumour suppressor role. These studied miRNAs may serve as minimally invasive biomarkers for BC and could represent promising potential therapeutic targets to increase patient survival and quality of life. However, validation and standardization of miRNA-based therapeutics are required to ensure their clinical efficacy.

## Supporting information

S1 Table Relation between miRNA 122,574, and 375 and clinicopathologic features of BC patients. (DOCX)

## List of abbreviations

BC: Breast cancer
miRNAs: MicroRNAs
