# Supplementary material for: Dysregulation of miR-122, miR-574 and miR-375 in Egyptian patients with breast cancer
Source: PLoS One. 2024 May 31;19(5):e0298536. doi: 10.1371/journal.pone.0298536 (PMC11142443; doi:10.1371/journal.pone.0298536)
Supplement: S1 Table — (DOCX) [file pone.0298536.s001.docx]

Supplementary table *(1):* Relation Between miRNA 122,574, and 375 and Clinicopathologic Features of BC Patients

| Mir 122 | | | | | Mir574 | | | | mir375 | | | |  | |
| --- | --- | --- | --- | --- | --- | --- | --- | --- | --- | --- | --- | --- | --- | --- |
| p value | >0.1875 | <=0.1875 | p value | | <=0.0139 | >0.0139 | p value | | <=2.5 | >2.5 | p value | |  |  |
| 0.09 | 23 | 15 | 0.42 | | 18 | 16 | 0.23 | | 15 | 20 | <=median | | Age |  |
|  | 18 | 21 |  |  | 23 | 20 |  |  | 17 | 25 | >median | |  |  |
| 0.67 | 2 | 0 | 0.55 | | 0 | 2 | 0.42 | | 1 | 1 | Negative | | ER |  |
|  | 50 | 25 |  |  | 24 | 51 |  |  | 56 | 19 | Positive | |  |  |
| 0.53 | 2 | 0 | 0.39 | | 0 | 2 | 0.86 | | 2 | 0 | Negative | | PR |  |
|  | 49 | 26 |  |  | 25 | 50 |  |  | 55 | 20 | Positive | |  |  |
| 0.46 | 47 | 18 | 0.73 | | 45 | 20 | 0.95 | | 48 | 17 | Negative | | HER2 |  |
|  | 5 | 7 |  |  | 8 | 4 |  |  | 9 | 3 | Positive | |  |  |
| 0.55 | 2 | 1 | 0.23 | | 1 | 2 | 0.38 | | 2 | 1 | 0 | | Grade |  |
|  | 3 | 2 |  |  | 3 | 3 |  |  | 3 | 2 | I | |  |  |
|  | 34 | 18 |  |  | 17 | 34 |  |  | 41 | 11 | II | |  |  |
|  | 4 | 13 |  |  | 15 | 2 |  |  | 12 | 5 | III | |  |  |
| 0.44 | 4 | 4 | 0.06 | | 6 | 2 | 0.66 | | 6 | 2 | stage1 | | Stage |  |
|  | 19 | 8 |  |  | 16 | 11 |  |  | 22 | 5 | I | |  |  |
|  | 23 | 11 |  |  | 28 | 6 |  |  | 24 | 10 | stage2 | |  |  |
|  | 7 | 1 |  |  | 0 | 8 |  |  | 7 | 1 | III | |  |  |
